# Bioactive Compounds, Antioxidant, Cytotoxic, and Genotoxic Investigation of the Standardized Liquid Extract from *Eugenia involucrata* DC. Leaves

**DOI:** 10.3390/ph18050764

**Published:** 2025-05-21

**Authors:** Monatha Nayara Guimarães Teófilo, Leonardo Gomes Costa, Jamira Dias Rocha, Fernando Gomes Barbosa, Anielly Monteiro de Melo, Grazzielle Guimarães de Matos, Cristiane Maria Ascari Morgado, Amanda Silva Fernandes, Lucas Barbosa Ribeiro de Carvalho, Clayson Moura Gomes, Milton Adriano Pelli de Oliveira, Joelma Abadia Marciano de Paula, Elisa Flávia Luiz Cardoso Bailão, Leonardo Luiz Borges

**Affiliations:** 1 Laboratory for Research, Development and Innovation of Biodiversity Products, State University of Goiás, Central Campus, Anápolis 75132-903, GO, Brazil; monathateofilo@gmail.com (M.N.G.T.); leonardogomescostaifg@gmail.com (L.G.C.); jamiradias@gmail.com (J.D.R.); fernandogomes.bio@gmail.com (F.G.B.); anielly_@hotmail.com (A.M.d.M.); cristiane.morgado@ueg.br (C.M.A.M.); joelma.paula@ueg.br (J.A.M.d.P.); elisa.flavia@ueg.br (E.F.L.C.B.); 2 Institute of Tropical Pathology and Public Health, Federal University of Goiás, Goiânia 74605-050, GO, Brazil; grazzi.guimaraes@gmail.com (G.G.d.M.); mapoliv@ufg.br (M.A.P.d.O.); 3 School of Medical and Life Sciences, Pontifical Catholic University of Goiás, Goiânia 74605-010, GO, Brazil; fer.amanda7@gmail.com (A.S.F.); lubarica@gmail.com (L.B.R.d.C.); claysonmoura@yahoo.com.br (C.M.G.); 4 Faculty of Military Principles, Goiânia 74120-020, GO, Brazil

**Keywords:** genoprotective effects, antioxidant activity, medicinal plants, Myrtaceae

## Abstract

**Background:** *Eugenia involucrata* DC., a Cerrado native plant, is recognized for its medicinal properties. However, its bioactive compounds remain inadequately explored. **Objectives**: This study investigated bioactive compounds from a standardized liquid extract from *E. involucrata* leaves that can act with antioxidant, cytogenotoxic, cytoprotective, and genoprotective effects. **Methods:** The phenolic compounds in the standardized liquid extract from *E. involucrata* leaves were screened by HPLC-DAD. The capture of the free radicals DPPH, ABTS^+^, and the metal reduction power FRAP determined the antioxidant potential. Cytotoxicity was evaluated in RAW 264.7 macrophages (MTT assay), and (anti)cytotoxic and (anti)genotoxic effects were assessed in human lymphocytes using the Trypan blue exclusion method and comet assay, respectively. **Results:** The extracts present key phenolic compounds, such as ellagic acid, myricitrin, and epicatechin gallate. The standardized extract demonstrated antioxidant capacity, evidenced by its ability to reduce iron and scavenge free radicals. The liquid extract from *E. involucrata* leaves exhibited cytotoxic effects on RAW 264.7 macrophages at higher concentrations, while demonstrating (anti)cytotoxic activity on human lymphocytes from all tested concentrations. The highest concentration tested of the standardized liquid extract from *E. involucrata* leaves (250 µg/mL) showed genotoxicity against human lymphocytes compared to the negative control. In contrast, the lowest concentration (62.5 µg/mL) exhibited an antigenotoxic effect on human lymphocytes, reducing the genotoxicity of doxorubicin by approximately 27%. **Conclusions:** The bioactive compounds in the standardized liquid extract from *E. involucrata* leaves exhibited antioxidant and antigenotoxic properties, suggesting potential value for nutraceutical and pharmaceutical applications, particularly those related to oxidative stress associated withaging and disease progression.

## 1. Introduction

*Eugenia involucrata* DC. is a native Brazilian species of the Myrtaceae family, known as “pitanga-do-cerrado” in the Cerrado biome. The leaves of this plant are popularly used as a therapeutic agent to treat diabetes mellitus [1]. Previous studies have demonstrated the presence of flavonoids, tannins, and terpenoids in the leaves, fruits, and seeds of *E. involucrata* [1,2]. The biological activity of essential oils and extract fractions from this species has also been described, indicating its therapeutic potential for various pathological conditions [1,2,3]. However, this species remains understudied regarding its phytochemical and pharmaceutical properties.

The leaves of *E. involucrata* and its extract showed the highest content of phenolic compounds, with ellagic acid, myricitrin, and epicatechin gallate being the most prominent [4,5]. These compounds exhibit various biological activities, including anti-inflammatory, hypoglycemic, antimicrobial, and antioxidant effects. Plant extracts with antioxidant properties can protect the body against oxidative stress in different cellular processes, such as delaying aging and preventing or reducing damage caused by disease mechanisms [6].

Oxidative stress is generated by the production of free radicals (reactive oxygen and nitrogen species), which are chemical entities that, in excess, cause cellular damage, including the destruction of the cell membrane and DNA [7]. Many diseases, such as obesity, hypertension, and cancer, involve oxidative stress due to excessive free radical production, which accelerates disease progression [8]. Species of the *Eugenia* genus, such as *Eugenia uniflora* L., have antiproliferative activity against gastric adenocarcinoma and modulate both cell viability and migration in melanoma tumor cells [9,10], highlighting the antitumor potential of the *Eugenia* genus.

Furthermore, the literature reports that compounds with antioxidant activity may reduce tumor cells’ incidence and progression by mitigating oxidative stress and/or delaying abnormal cell division [11,12,13]. According to the Pan American Health Organization, cancer is one of the leading causes of death in the Americas. Additionally, it is estimated that by 2045, there will be six million cases, alarming data for public health [14]. Species of the *Eugenia* genus show significant antioxidant potential due to their phenolic compounds, such as flavonoids and tannins. These compounds effectively scavenge free radicals, thereby reducing oxidative stress associated with aging and various diseases. Supported by studies, the antioxidant properties of *Eugenia* spp. suggest their use in developing natural therapies to prevent cellular damage and enhance health, with potential applications in traditional and modern medicine [15,16].

The safety of using medicinal plants and herbal products is essential for ensuring consumer health and well-being. While many plants have traditional therapeutic benefits, they may also pose risks, including toxicity, drug interactions, and adverse effects. Rigorous safety evaluations help identify safe dosages, potential toxicities, and contraindications, enabling informed and responsible use by healthcare professionals and patients. Promoting safe practices in herbal medicine builds public trust and encourages the informed application of these alternatives in modern healthcare in harmony with cultural traditions [17,18].

Therefore, this study aimed to investigate the biological properties of *Eugenia involucrata* leaves by characterizing and standardizing the liquid extract, evaluating its antioxidant activity through DPPH (2,2-diphenyl-1-picrylhydrazyl), ABTS^+^, and FRAP (ferric reducing antioxidant power) assays, and determining its cytotoxicity in RAW 264.7 murine macrophages, as well as its cytogenotoxic, cytoprotective, and genoprotective effects on human lymphocytes.

## 2. Results and Discussion

### 2.1. Physicochemical Characterization of the Liquid Extract

Characterizing the liquid extract is essential to establishing process standardization controls, ensuring medicinal properties’ quality, safety, and reproducibility [19]. These parameters also serve as a foundation for technological development in herbal products [20]. The liquid extract exhibited a pH of 5.35 ± 0.01, a solid content of 4.59 ± 0.30%, and a density of 1.03 ± 0.02. The acidic pH enhances the stability of bioactive compounds and inhibits microbial growth [21], indicating the presence of acidic constituents. Density and solid content are critical for quality assessment and guiding downstream technological processes, such as drying [22].

Phenolic compounds are key markers for evaluating plant extracts’ biological potential. The liquid extract contained 1.5 ± 0.11 mg/mL of phenolics and 0.63 ± 0.01 mg/mL of flavonoids. Using the modified Rufino and Alves (2007) method [23], polyphenol content was quantified at 700.9 ± 6 mg gallic acid/100 g, corroborating prior results via an alternative methodology. This value surpassed those reported for other Brazilian species: *Eugenia dysenterica* (201.1 ± 10.7 mg/100 g), *Byrsonima crassifolia* (259.2 ± 13.8 mg/100 g), and *Annona montana* (192.1 ± 17.3 mg/100 g) [23,24].

### 2.2. Screening of Phenolic Compounds in Liquid Extract Obtained by Ultrasound from Eugenia involucrata Leaf Powder and Liquid Extract by HPLC-DAD

**Figure 1:**
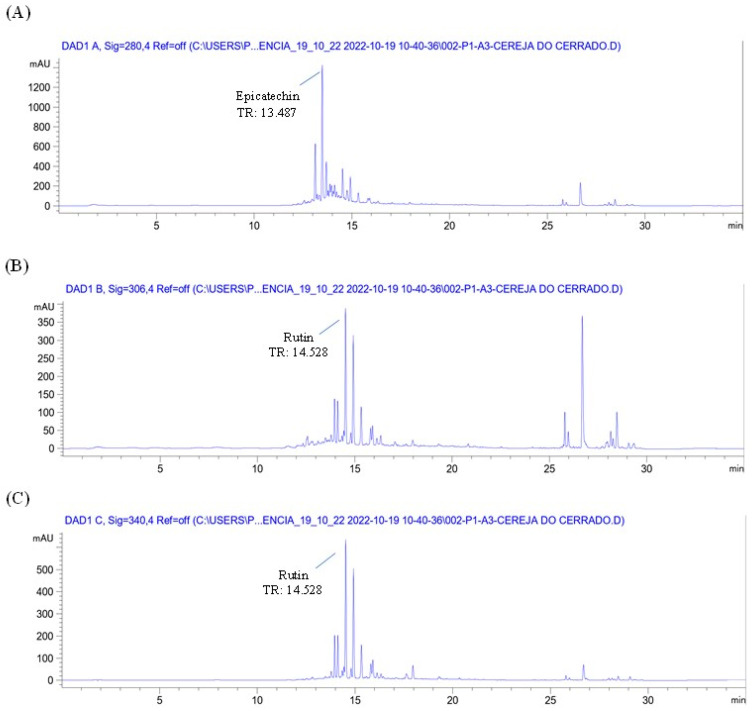
Chromatogram of the plant drug (dried and powdered leaves) of *Eugenia involucrata* at 280 nm (**A**), 306 nm (**B**), and 340 nm (**C**) by HPLC-DAD. Extract obtained by ultrasound-assisted extraction. Chromatographic conditions: The analysis used a mobile phase of acetonitrile and water acidified with 0.2% acetic acid in a gradient elution system. The method employed an injection volume of 5 μL, a 1 mL/min flow rate, and maintained the column temperature at 30 °C. Detection was achieved using a diode array detector (DAD) with a C18 column. Peak heights were recorded in milliabsorbance units (mAU), and retention times in minutes (min).

**Figure 2:**
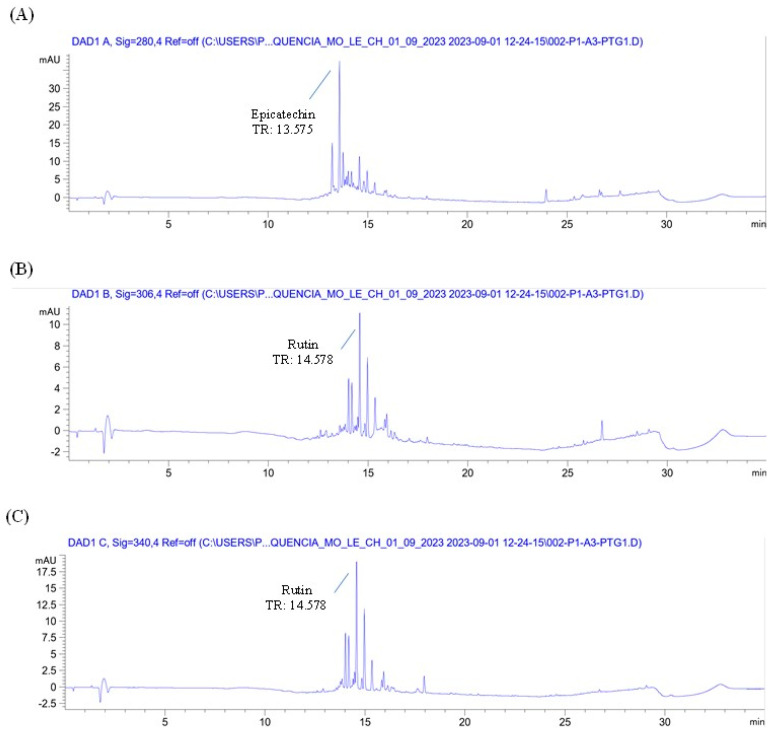
Chromatogram of the liquid extract from *Eugenia involucrata* leaves at 280 nm (**A**), 306 nm (**B**), and 340 nm (**C**) by HPLC-DAD. Chromatographic conditions: Mobile phase consisting of acetonitrile and water acidified with 0.2% acetic acid, using gradient elution. Injection volume: 5 μL; flow rate: 1 mL/min; column temperature: 30 °C. Diode array detector (DAD); C18 column. Peak height (mAU); time in minutes (min); retention time (RT).

**Figure 3:**
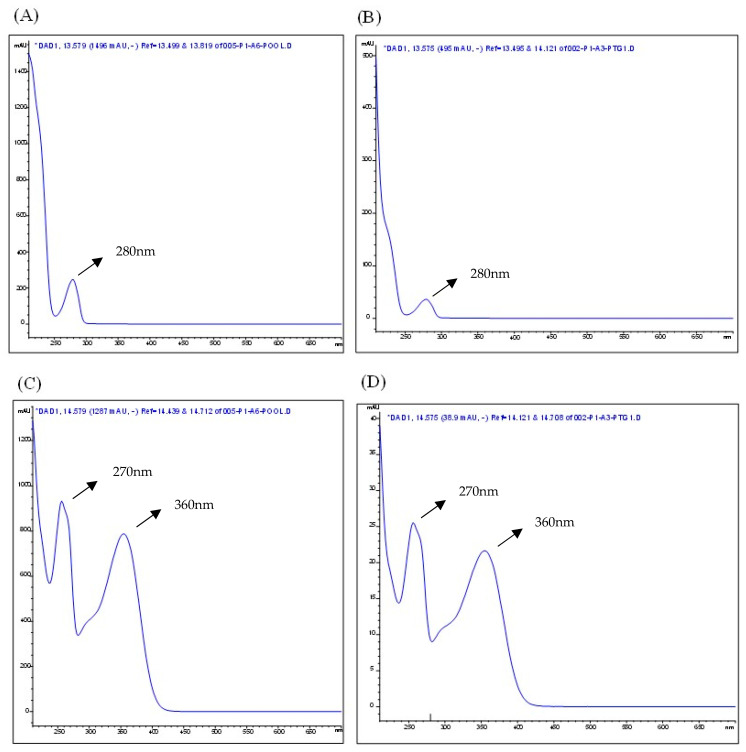
The absorption spectra in the Ultraviolet–Visible (UV–Vis) region correspond to the peaks of the epicatechin standard (**A**) and epicatechin in the liquid extract from *Eugenia involucrata* leaves (**B**), as well as the rutin standard (**C**) and rutin in the liquid extract from *E. involucrata* leaves (**D**), as analyzed by HPLC-DAD. Chromatographic conditions: The analysis used a mobile phase composed of acetonitrile and water acidified with 0.2% acetic acid, with gradient elution. The method employed an injection volume of 5 μL, a 1 mL/min flow rate, and maintained the column temperature at 30 °C. Detection was performed using a diode array detector (DAD), with separation achieved on a C18 column. *DAD: Diode Array Detection.

The chromatographic profile of the plant material (*Eugenia involucrata* leaves) and analytical standards are shown in Figure 1 and Supplementary Figure S1, respectively. The chromatographic analysis of the plant material indicated the presence of two phenolic compounds: epicatechin, detected at 280 nm with a retention time (RT) of 13.487 min, and rutin, detected at 340 nm with an RT of 14.528 min (Figure 1A,C). These findings were confirmed by comparing the retention times with those of the analytical standards for epicatechin (RT: 13.495 min) and rutin (RT: 14.529 min) (Supplementary Figure S1A,C). The liquid extract also contained rutin and epicatechin, as shown in Figure 2 and Figure 3.

A previous study also detected the presence of epicatechin and rutin in *E. involucrata* leaves [25]. However, those authors reported additional compounds, such as ellagic acid, gallic acid, quercetin, and catechin, which were not detected in our study. The absence of these compounds may be attributed to several factors, including the detection limit of the equipment and co-elution with other compounds. Thus, modifications in sample preparation techniques or analytical methods could enhance their detectability [26]. The absence of ellagic acid and other related compounds in our results may be attributed to our study’s specific extraction method and chromatographic conditions. Different extraction techniques, solvents, and chromatographic parameters can influence the profile of detected compounds, potentially leading to the non-detection of certain phenolics such as ellagic acid, which are commonly reported in other *Eugenia* species [27,28,29].

Epicatechin is a flavonoid belonging to the flavan-3-ol class, characterized by hydroxyl groups at positions 3′ and 4′ on the B ring. It has been associated with various biological activities, including antioxidant, antimicrobial, anti-inflammatory, antitumor, and cardioprotective effects [30]. Rutin (quercetin-3-rutinoside) is another flavonoid, classified as a flavonol, consisting of an aglycone (quercetin) bound to a rutinoside disaccharide. The literature reports highlight its multiple biological activities, such as analgesic, antiarthritic, antibacterial, cardioprotective, antioxidant, and antidiabetic properties [31]. Both compounds have been previously reported in species of the *Eugenia* genus [25].

### 2.3. Determination of the Antioxidant Activity of the Liquid Extract

The antioxidant activity of the *E. involucrata* leaf extract was evaluated by DPPH, ABTS^+^, and the metal reduction power FRAP (Table 1). The ABTS^+^ method, applicable to hydrophilic and lipophilic matrices [32,33], showed the highest activity (2701.67 μM Trolox/g extract), followed by FRAP (1490.79 μM Fe^2+^/g extract), which evaluates the electron-donating capacity with high reproducibility [33]. The DPPH assay, based on hydrogen or electron transfer to a stable radical [32], indicated moderate activity (75.0 ± 11 µg extract/µg DPPH). These differences highlight the extract’s broad antioxidant profile and the relevance of combining multiple assays for a more comprehensive evaluation.

In this context, relying on a single method to determine antioxidant activity may yield insufficient information due to multiple factors, including the type of matrix used and the different mechanisms/sites of antioxidant action [34]. Therefore, employing various methods, as presented in this study, becomes essential to provide a more comprehensive understanding of the potential mechanisms of the analyzed material.

Compared with other species of the same genus evaluated in previous studies, the leaf extract of *E. involucrata* demonstrates a promising antioxidant profile. For instance, in the study by Ferreira et al. (2023) [35], *Eugenia uniflora* exhibited a lower DPPH IC_50_ value (1.46 µg/mL), indicating more vigorous radical scavenging activity in that specific assay. Conversely, its ferric reducing antioxidant power (FRAP) value was significantly lower (78.45 µM TE/g), suggesting that *E. involucrata* possesses superior ferric ion-reducing capacity.

Barbosa et al. (2025) [15] reported that *Eugenia dysenterica* showed FRAP values between 1633 and 1692 µM FeSO_4_/g and DPPH activity between 73 and 83 g sample/g DPPH. These values are close to those obtained for *E. involucrata*, indicating a similar antioxidant capacity. Notably, the ABTS^+^ activity of *E. involucrata* (2701.67 μM Trolox/g extract) exceeds the values observed for *E. dysenterica* (1379 to 1633 μM Trolox/g), suggesting a higher capacity for radical scavenging through electron transfer mechanisms [15]. Moreover, phenolic compounds, including epicatechin and rutin identified in this extract, exhibit potent antioxidant activity due to their ability to scavenge free radicals, donate hydrogen/electrons, and chelate transition metals [33]. Their presence substantiates the extract’s potential for antioxidant applications.

Other species of the genus *Eugenia* demonstrated antioxidant activity using the DPPH, ABTS^+^, and FRAP methods. The essential oil from the leaves of *Eugenia uniflora* L. exhibited antioxidant activity in DPPH, ABTS^+^, and FRAP assays [36]. The antioxidative activity of essential oils from the stem (0.82 ± 0.15 μg/mL), bud (1.18 ± 0.56 μg/mL), and leaf (1.16 ± 0.74 μg/mL) of *Eugenia caryophylata* was also demonstrated by the DPPH method and ABTS^+^ method with values for the stem (0.81 ± 0.16 μg/mL), bud (0.54 ± 0.77 μg/mL) (0.81 ± 0.16 μg/mL), and leaves (0.66 ± 0.67 μg/mL) [37]. The leaf extract of *Eugenia punicifolia* was also able to demonstrate antioxidant activity by DPPH (28.84 ± 0.54 μg/mL) and ABTS (10.5 ± 1.2 μg/mL) [38]. The species *Eugenia stipitata* also showed radical scavenging activity, with a DPPH IC_50_ of 0.69 ± 0.23 μg/mL [39]. The standard of rutin was employed as a positive control in all the methodologies, and the values obtained were 228.4 g standard/g DPPH, 2047.3 μM ferrous sulfate/g rutin, and 1410.4 μM Trolox/g extract. Regarding the positive control rutin, the standardized liquid extract of *E. involucrata* demonstrated superiority compared to the DPPH and ABTS^+^ methods. This suggests that the extract contains a complex mixture of phenolic compounds and flavonoids that may synergistically enhance its radical scavenging capacity. These findings support the possible application of this extract in protecting against oxidative stress-related conditions and warrant further investigation into the specific constituents responsible for this activity.

### 2.4. Cytotoxic Investigation in Murine Macrophages

**Figure 4:**
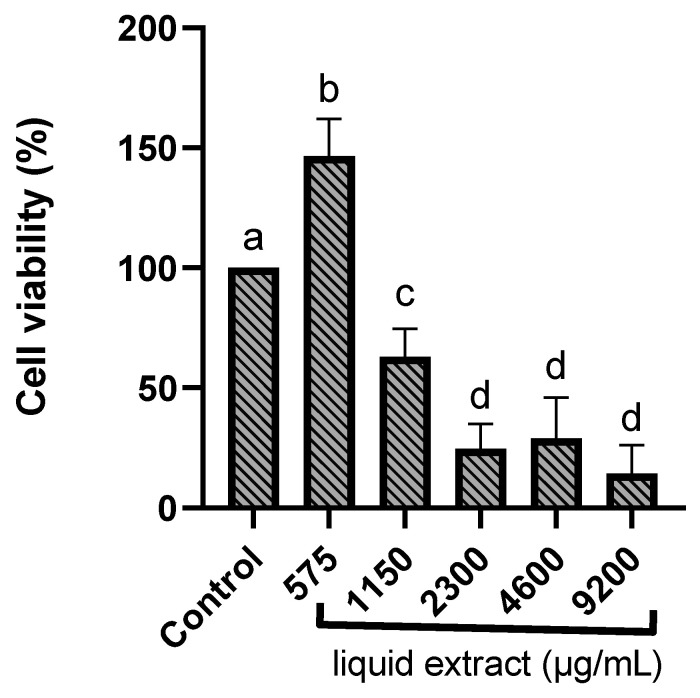
Cytotoxicity of the standardized liquid extract from *Eugenia involucrata* leaves on RAW 264.7 murine macrophages. Quantification of RAW 264.7 murine macrophage viability in the presence of different concentrations of *Eugenia involucrata* (575; 1150; 2300; 4600; and 9200 μg/mL) and negative control (non-treated cells) using an MTT assay. Data are shown as mean ± standard deviation (SD) and were evaluated by one-way ANOVA. After multiple comparisons of means using the Tukey test, all treatments were statistically different, except for the treatments 2300 vs. 4600, 2300 vs. 9200, and 4600 vs. 9200; a, b, c, and d represent the different groups formed after multiple comparisons. Significant difference in comparison with the control (*p* < 0.05).

The MTT colorimetric assay assessed the viability of RAW 264.7 murine macrophages incubated with *E. involucrata* extract. Cells were treated with different concentrations of the extract to determine potential cytotoxicity. The liquid extract from *E. involucrata* leaves demonstrated cytotoxicity at higher concentrations, ranging from 1110 to 9200 µg/mL, in RAW 264.7 murine macrophages. The calculated IC_50_ value for the liquid extract of *E. involucrata* was 1468.51 ± 143.34 µg/mL. The cell viability data at each concentration are shown in Table S1. Gupta et al. (2002) showed that polyphenols can initiate auto-oxidation and produce toxicity in high concentrations [40]. However, no toxic effects were observed at a 575 µg/mL concentration (cell viability above 80%) (Figure 4), suggesting that the extract is safe at this concentration. Moreover, an increase in cell viability was observed at this concentration. Similarly, the methanol extract of *Calophyllum brasiliense* has been shown to promote increased cell proliferation using the MTT assay [41]. Furthermore, it has been demonstrated that plant extracts can stimulate cell proliferation due to their antioxidant activity [42].

These results highlight the importance of cytotoxicity assays in evaluating the safety of plant-derived extracts. Macrophages, as the primary resident phagocytic cells in all tissues, play a vital role in clearing waste materials from the blood and maintaining homeostasis, making them a relevant model for toxicity studies [34]. Cytotoxicity assays represent a valuable tool for the preliminary safety assessment of medicinal plants as they reveal potential toxic effects on various cell types, including human cells. This information guides using plant-based remedies, ensuring therapeutic benefits without health risks. These evaluations support the development of effective and safe natural therapies for modern healthcare [43,44].

### 2.5. Cytogenotoxic Evaluation of the Liquid Extract from Eugenia involucrata Leaves

Based on the results obtained in the RAW 264.7 macrophage model, the concentrations selected for evaluation in human lymphocytes were lower than the highest non-toxic dose identified in the murine macrophage assay. This approach was adopted to ensure cellular integrity in primary human cells, which may be more sensitive than immortalized cell lines, and to avoid potential false-positive results in genotoxicity testing due to cytotoxic interference [45,46]. Moreover, using sub-toxic concentrations is recommended in genotoxic and genoprotective assays to distinguish better direct DNA effects from secondary damage caused by cytotoxicity [47].

**Figure 5:**
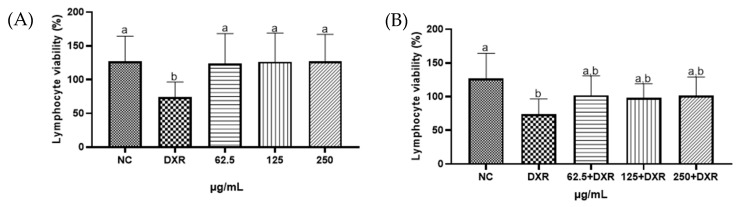
Viability of human lymphocytes treated with the standardized liquid extract from *Eugenia involucrata* leaves (62.5; 125; and 250 µg/mL) in the absence (**A**) or presence (**B**) of doxorubicin (DXR). A negative control group (NC; sterile water) was also included. Different letters indicate statistically significant differences between groups (*p* < 0.05), whereas the same letters indicate no significant difference (*p* > 0.05); a and b represent the different groups formed after multiple comparisons.

In the present study, the Trypan blue assay was employed to assess the cytotoxic potential of the liquid extract of *E. involucrata* leaves. The results demonstrated that the extract exhibited no cytotoxicity against human lymphocytes at the concentrations tested in this study (Figure 5A, *p* > 0.05). When co-treated with doxorubicin (DXR), the liquid extract did not exhibit cytoprotective effects (*p* > 0.05), nor did it enhance the cytotoxic effect of DXR on human lymphocytes at the tested concentrations (Figure 5B, *p* > 0.05). As expected, DXR caused a significant decrease in cell viability compared to the control group (Figure 5A,B, *p* < 0.05) [48]. Similarly, cytotoxicity was absent for the leaf extract of *Eugenia uniflora*, a species of the same genus of *E. involucrata* (Cunha et al., 2016 [49]). However, the crude ethanolic extract of *E. involucrate* seeds showed cytotoxicity (1000 mg·mL^−1^) in non-cancerous cells (human umbilical vein endothelial cells—HUVEC, and monkey kidney epithelial cells—VERO). The fruit extract was also investigated but did not exhibit such activity [50].

**Figure 6:**
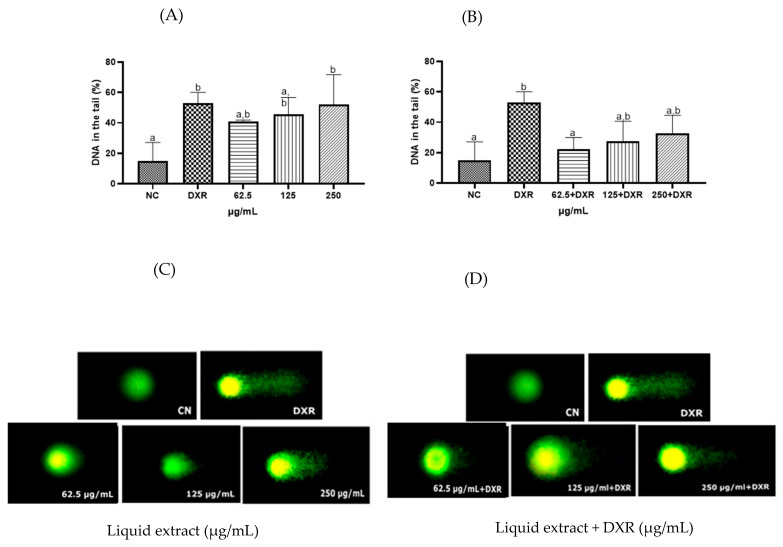
Effect of the standardized liquid extract from *Eugenia involucrata* leaves on DNA of human lymphocytes in the absence (**A**,**C**) or presence (**B**,**D**) of doxorubicin. Negative control (NC, sterile water); doxorubicin (DXR, 50 µg/mL). The values are expressed as the mean (± standard deviation) of three independent experiments, *p* < 0.05; a and b represent the groups formed after multiple comparisons. Cells were stained with the nucleic acid dye Diamond™, and images were captured using a fluorescence microscope (Axioplan-ImagingVR), with Zen Blue Software 2.3, using an excitation filter of 510–560 nm and a barrier filter of 590 nm (10× objective).

Regarding the genotoxicity assessment, the standardized liquid extract of *E. involucrata* leaves exhibited genotoxicity against human lymphocytes at the highest concentration tested (250 µg/mL) when compared to the negative control, as detected by the comet assay (Figure 6A, *p* < 0.05). On the other hand, a genoprotective effect was observed at the lowest concentration tested of the standardized liquid extract of *E. involucrata* (62.5 µg/mL), which reduced the genotoxicity of doxorubicin by approximately 27% (Figure 6B, *p* < 0.05). As expected, DXR (positive control) caused significant DNA damage compared to the negative control cells (Figure 6A,B; *p* < 0.05).

The genotoxicity of our species differs from the results obtained by Cunha et al. (2016) [49] for *E. uniflora*, whose leaf extract showed the absence of both cytotoxic and genotoxic effects even at higher concentrations (240 and 480 µg/mL). This result was attributed to compounds such as quercetin, quercitrin, isoquercitrin, luteolin, and ellagic acid [49], which were not detected in our extract using the methods employed. Furthermore, other unidentified compounds in the extract (due to the techniques used in this study) may lead to a genotoxic effect at higher concentrations, as observed at 250 µg/mL. The results presented by Neri-Numa et al. (2013) demonstrated that the ethanolic extract of *Eugenia stipitata*, a species of the same genus, presented antimutagenic and genoprotective properties at the highest concentration tested (300 mg/kg of body weight) [39].

On the other hand, under the conditions of this study, we identified the presence of epicatechin and rutin in the liquid extract of *E. involucrata*. These flavonoids are known for their antioxidant potential, which helps capture and eliminate radicals. They are among medicinal plants’ most widely found secondary metabolites [51,52]. Evidence has shown that compounds with antioxidant properties can remove ROS before these species interact with the DNA molecule, resulting in permanent damage [53]. Studies suggest chemoprotective activities are associated with the antioxidant capacity of these metabolites [54]. Khan et al. (2018) found that rutin solutions at 20 µg/mL and 40 µg/mL could reduce in vitro genetic damage induced by methyl methanesulfonate in human lymphocytes by 20.57% and 47.14%, respectively [55].

In general, rutin, epicatechin, and phenolic compounds are known for their powerful antioxidant properties, which are crucial in protecting cells from oxidative damage and genotoxic stress. These bioactive molecules can neutralize free radicals and reactive oxygen species, reducing oxidative stress that can lead to DNA damage, mutations, and cell death. Additionally, phenolic compounds such as antioxidant enzymes may enhance the activity of cellular defense systems and inhibit pathways involved in inflammation and apoptosis. Their ability to modulate cellular responses helps provide genoprotective effects, contributing to the maintenance of genomic stability and overall cell integrity. This protective mechanism is critical in conditions associated with oxidative and genotoxic stress, highlighting the potential therapeutic benefits of these compounds in preventing cellular damage [56,57,58].

Using the comet assay, Haza and Morales (2011) [59] evaluated the protective effect of epicatechin (10–50 µM) against oxidative DNA damage induced by carcinogens in HepG2 cells. Their findings indicated that epicatechin significantly reduced lesions caused by N-nitrosodibutylamine and N-nitrosopiperidine. Moreover, the authors observed that epicatechin attenuated oxidative lesions promoted by N-nitrosodibutylamine and N-nitrosopiperidine in the genetic material of the cell line [48,59].

In line with these findings, the standardized extract of *E. involucrata* exhibited a significant protective effect against DXR genotoxicity at 62.5 µg/mL against DXR-induced damage. DXR, a recognized antitumor drug, induces DNA breaks and generates free radicals, producing cellular damage [60,61]. The DXR molecule can also directly interact with iron to form a DXR–Fe complex, facilitating iron cycling between Fe^2+^ and Fe^3+^ forms, producing significant ROS, and causing cellular damage [62].

The metal-chelating capacity of several phenolic compounds and their antioxidant activities may account for the genoprotective effects of the liquid extract observed in this study. Supporting this hypothesis, the extract demonstrated significant antioxidant activity, as evidenced by its free radical scavenging and ferric-reducing capabilities. These properties underscore its potential to attenuate oxidative stress and DNA damage caused by genotoxic agents. Furthermore, antioxidant compounds in the extract, such as epicatechin and rutin, may have played a protective role against genetic damage induced by doxorubicin.

Compounds lacking genotoxicity might be used for therapeutic purposes with reduced concern for DNA-related adverse effects. Further, the genoprotective properties of a substance indicate its potential for preventing DNA damage, which may be particularly relevant in treatments to reduce the side effects of chemotherapeutic agents [63,64]. The phenolic compounds, including rutin and epicatechin, possess notable antioxidant and genoprotective properties that could significantly contribute to developing novel therapies for genotoxic conditions. By effectively neutralizing reactive oxygen species and reducing oxidative stress, these compounds may protect DNA integrity and prevent mutations induced by environmental agents, radiation, or chemotherapeutic drugs. Their ability to modulate cellular defense mechanisms suggests they could be used as adjuncts alongside conventional treatments to minimize collateral DNA damage in healthy cells, thereby reducing side effects and enhancing overall treatment efficacy [65,66]. Future therapeutic strategies might involve using the *E. involucrata* extract or its bioactive compounds to develop protective agents or supplements that shield normal tissues during genotoxic exposures. Thus, future studies are necessary to investigate the compounds involved in DNA damage and the protective effects of *E. involucrata* extract in the present study.

## 3. Materials and Methods

### 3.1. Plant Material

The leaves of *Eugenia involucrata* were collected in the municipality of Hidrolândia (latitude 16°54′01.0″ S, longitude 49°15′32.5″ W, 832 m altitude) located in the State of Goiás, Brazil. Prof. Dr. José Realino de Paula identified the specimen from the Federal University of Goiás, and the herbarium material was deposited as a voucher at the Herbarium of the State University of Goiás (UEG-GO) under the voucher number 15,366 (Figure S1). After collection, the leaves were dried in an oven at 40 °C with air circulation, and the resulting material was ground in a knife mill, weighed, and stored in a container protected from light and moisture. The nomenclature of the species was reviewed in Index Plant Names. The post-taxonomic revision nomenclature of the section Phyllocalyx (homotypic) was adopted in this research, from *Eugenia calycina* Cambess to *Eugenia involucrata* DC., as stated in the Taxonomic Catalog Flora and Funga of Brazil (2020) and the description of the original type specimen in Fl. bras. Merid. 2: 352 (1832) [67].

### 3.2. Screening of Phenolic Compounds in Liquid Extract Obtained by Ultrasound from Eugenia involucrata Leaf Powder by HPLC-DAD

In a 25 mL volumetric flask, 1 g of the plant sample (powdered) and 10 mL of 60% ethanol (*w*/*w*) were added. The flask was sonicated in an ultrasonic bath (Ultronique model Q5.9/40A, frequency 40 kHz, power 200 W) at 60 °C for 30 min. After this period, the solution was subjected to simple filtration using filter paper. From the filtrate, 2 mL was transferred to an Eppendorf tube and centrifuged (IKA^®^ mini G) at 6000 rpm for 15 min. Subsequently, the supernatant was filtered again, using a PTFE membrane (0.45 μm), and collected in a 2 mL amber vial before being used for phenolic compound screening.

The screening was performed using HPLC-DAD (model G7115A) with the OpenLab CDS software 2.8 and an autosampler (Agilent Technologies, Inc. 1290 Infinity II, Santa Clara, CA, USA). Chromatographic separations were carried out using an Agilent InfinityLab Poroshell 120 EC-C_18_ column (4.6 × 100 mm, 2.7 µm) with a mobile phase consisting of acetonitrile and water acidified with 0.2% acetic acid in a gradient elution (Table 1). The analysis was conducted at a 1 mL/min flow rate with an injection volume of 5 µL and a column temperature maintained at 30 °C. Detection was performed at three different wavelengths (280 nm, 306 nm, and 340 nm) to identify phenolic compounds by comparing their retention times with analytical standards. For this study, ten reference standards from Sigma-Aldrich (St. Louis, MO, USA) were used, all prepared in HPLC-grade methanol: resveratrol (0.2% *w*/*v*), gallic acid (0.2% *w*/*v*), caffeic acid (0.2% *w*/*v*), ellagic acid (0.2% *w*/*v*), quercetin (0.2% *w*/*v*), catechin (0.2% *w*/*v*), epicatechin (0.2% *w*/*v*), rutin (0.2% *w*/*v*), apigenin (0.2% *w*/*v*), and kaempferol (0.08% *w*/*v*) (Supplementary Figure S2).

The powdered plant material (50 g) was placed in a suitable container and uniformly moistened with 54% (*w*/*w*) ethanol. The container was then kept at rest, protected from light, for 24 h, to allow swelling of the material. After this period, the sample was transferred to a stainless-steel percolator and left for maceration for 3 days. The percolation was performed exhaustively for 5 days using 750 mL of 57% (*w*/*w*) ethanol. The extract was then concentrated by rotary evaporation (IKA^®^ RV10) under controlled conditions of a 40 °C temperature, 25 rpm rotation speed, and 80 bar pressure to obtain the final concentrated extract.

### 3.3. Characterization of the Concentrated Liquid Extract from Eugenia involucrata Leaves

The physicochemical characterization of the concentrated liquid extract from *Eugenia involucrata* leaves was performed through three key analyses, pH determination, solid content measurement, and density evaluation, all conducted in triplicate according to standardized methodologies [19]. The pH was measured using a calibrated Tecnopon pH meter, with prior calibration using pH 4.0 and 7.0 buffer solutions to ensure accuracy. For solid content determination, 0.5 g samples of the extract were evenly distributed on the balance plate of a MOC-63U SHIMADZU (Kyoto, Japan) instrument equipped with a halogen heating lamp and heated at 105 °C until reaching constant weight, with results calculated as 100% minus the moisture percentage. Density was assessed through a 10 mL pycnometer, where the empty, clean, and dry pycnometer was first weighed, then filled with distilled water at 20 °C and weighed again, followed by the same procedure with the extracted sample. The mass density was calculated using the formula that accounts for the sample’s relative density compared to water and a standard correction factor of 0.0012, providing precise density values in g/mL. These comprehensive analyses established fundamental quality parameters for the extract, ensuring reproducibility and standardization for potential applications.

### 3.4. Quantification of Total Phenolics and Flavonoids

The quantitative analysis of total phenolic compounds was conducted according to the spectrophotometric method of Hagerman and Butler (1978), as modified by Mole and Waterman (1987) [68,69]. A stock standard solution at 0.1 mg/mL in distilled water was prepared, from which a series of dilutions (0.050, 0.100, 0.150, 0.200, 0.250, and 0.300 mg/mL) were made to establish the calibration curve. The assay mixture consisted of 2 mL of sodium lauryl sulfate–triethanolamine solution (SLS 1% *w/v*–TEA 5% *v*/*v*), 1 mL of ferric chloride solution (1.62 g/L in 0.001 M hydrochloric acid), and 1 mL of sample or standard solution. After a 15 min incubation period, absorbance readings were taken at 510 nm using a Metash ESPEC-UV-5100 UV–Vis (Shanghai, China) spectrophotometer. The plant extract was diluted 50-fold in distilled water for sample analysis, and the same reaction conditions were applied. All measurements were performed in triplicate, with results expressed as mean values ± standard deviation in mg/mL.

A modified version of the Rolim et al. (2005) method was employed for flavonoid quantification [70]. A rutin standard solution (0.1 mg/mL in methanol–0.02 M acetic acid 99:1) served as the reference, with subsequent dilutions ranging from 0.005 to 0.025 mg/mL prepared for calibration. The plant extract was diluted 200-fold and mixed with the methanol–acetic acid solvent to a final volume of 4 mL. Absorbance measurements were conducted at 361 nm using the exact spectrophotometer, with all analyses performed in triplicate. The blank consisted of the methanol–acetic acid solvent alone. Final flavonoid concentrations were calculated based on the rutin standard curve and reported as mean ± standard deviation in mg/mL. Both analytical procedures included appropriate blank controls and were conducted under standardized conditions to ensure the reproducibility of results.

### 3.5. Determination of Phenolic Compounds in Liquid Extract of Eugenia involucrata by HPLC-DAD

The phenolic compounds present in the concentrated liquid extract of *Eugenia involucrata* were analyzed using HPLC-DAD (Agilent Technologies 1290 Infinity II system). Chromatographic separation was achieved using an Agilent InfinityLab Poroshell 120 EC-C18 column (4.6 × 100 mm, 2.7 µm) with the following parameters: a mobile phase flow rate of 1 mL/min, column temperature maintained at 30 °C, and injection volume of 5 µL. The mobile phase consisted of acetonitrile and water acidified with 0.2% acetic acid, applied in a gradient elution (Table 2). Detection was performed at three wavelengths (280 nm, 306 nm, and 340 nm) for comprehensive compound identification.

Analytical standards from Sigma-Aldrich were prepared in HPLC-grade methanol at the following concentrations: resveratrol (0.2% *w*/*v*), gallic acid (0.2% *w*/*v*), caffeic acid (0.2% *w*/*v*), ellagic acid (0.2% *w*/*v*), quercetin (0.2% *w*/*v*), catechin (0.2% *w*/*v*), epicatechin (0.2% *w*/*v*), rutin (0.2% *w*/*v*), apigenin (0.2% *w*/*v*), and kaempferol (0.08% *w*/*v*).

For sample preparation, 1 mL of concentrated liquid extract was transferred to a 10 mL volumetric flask and diluted to volume with HPLC-grade methanol. The solution was sonicated for 15 min at room temperature using an ultrasonic bath (Ultronique model Q5.9/40A, 40 kHz frequency, 200 W power), followed by filtration through filter paper. A 2 mL aliquot of the filtrate was centrifuged at 6000 rpm (IKA mini G; Staufen, Germany) for 15 min, and the supernatant was filtered through a 0.45 µm PTFE membrane into a 2 mL amber vial before analysis.

Compound identification was based on retention time matching and comparison of UV–Visible absorption spectra between the analytical standards and corresponding peaks in the plant extract. This dual confirmation approach (retention time and spectral data) ensured the accurate identification of phenolic compounds in the sample. The analytical process was conducted under controlled conditions to maintain method reproducibility and reliability.

The analytical method was validated for the simultaneous quantification of rutin and epicatechin, evaluating the parameters of selectivity, linearity, matrix effect, precision, accuracy, robustness, limit of detection (LOD), and limit of quantification (LOQ), in compliance with the requirements of Resolution RDC No. 166/2017 from the Brazilian Health Regulatory Agency (Anvisa) and the Association of Official Analytical Chemists International [71,72].

### 3.6. Determination of Antioxidant Activity of the Liquid Extract

#### 3.6.1. Sample Preparation

The sample preparation for the following methods (FRAP, DPPH, and ABTS^•+^) was performed according to the methodology adapted from Larrauri, Rupérez, and Saura-Calixto [73]. The positive control used for all the analyses was the standard of rutin (Sigma Aldrich^®^).

#### 3.6.2. Ferric Reducing Antioxidant Power (FRAP) Method

The total antioxidant activity was determined using the FRAP assay according to the modified method of Rufino et al. (2006) [23]. A standard solution was prepared by dissolving 27.8 mg of ferrous sulfate in distilled water and adjusting the volume to 50 mL in a volumetric flask. This solution was homogenized and transferred to a labeled amber glass vial. From this stock solution, standard curve concentrations were prepared: 500 μM, 1000 μM, 1500 μM, and 2000 μM [23]

For the assay, 90 μL aliquots of each ferrous sulfate solution (in duplicate) were transferred to test tubes, followed by adding 270 μL of distilled water and 2.7 mL of FRAP reagent. The tubes were homogenized and kept in the dark for 30 min. The blank consisted of the FRAP reagent alone.

For the liquid extract evaluation, five dilutions were prepared from Solution A (4000 mg/L, described in Section 3.6.1.), yielding concentrations of 1000 mg/L, 760 mg/L, 500 mg/L, 248 mg/L, and 124 mg/L. Aliquots (90 μL) of each dilution (in duplicate) were mixed with 270 μL of distilled water and 2.7 mL of FRAP reagent. After 30 min of incubation in the dark, absorbance was measured at 595 nm using a UV–Vis spectrophotometer (Metash, model ESPEC-UV-5100; Shanghai, China). Results were expressed as μM ferrous sulfate/g extract.

#### 3.6.3. ABTS^+^ Radical Scavenging Method

The total antioxidant activity was determined using the ABTS^+^ radical method, following the modified protocol of Rufino et al. (2007) [74]. A standard solution was prepared by dissolving 25 mg of Trolox in ethanol and adjusting the volume to 50 mL in a volumetric flask. This solution was homogenized and stored in a labeled amber vial. Standard curve concentrations (100 μM, 500 μM, 1000 μM, 1500 μM, and 2000 μM) were prepared from this stock [74].

For the assay, 30 μL aliquots of each Trolox dilution (in duplicate) were mixed with 3 mL of ABTS^•+^ radical solution. After homogenization, absorbance was measured at 734 nm after 6 min of incubation in the dark. The blank consisted of ethanol.

For the liquid extract, five dilutions were prepared from Solution A (4000 mg/L), yielding concentrations of 1000 mg/L, 760 mg/L, 500 mg/L, 248 mg/L, and 124 mg/L. Absorbance was measured at 734 nm (Metash ESPEC-UV-5100; (Shanghai, China). Results were expressed as μM Trolox equivalents/g extract.

#### 3.6.4. 2,2-Diphenyl-1-picrylhydrazyl (DPPH) Radical Scavenging Method

The total antioxidant activity was determined using the DPPH radical method, following the modified protocol of Rufino et al. (2007) [74]. A standard solution was prepared by dissolving 2.4 mg of DPPH in methanol and adjusting the volume to 100 mL in a volumetric flask. This solution was homogenized and stored in a labeled amber vial. Standard curve concentrations (10 μM, 20 μM, 30 μM, 40 μM, 50 μM, and 60 μM) were prepared from this stock [75].

For the assay, 4 mL aliquots of each DPPH solution were transferred to test tubes, homogenized, and measured at 515 nm (Metash ESPEC-UV-5100; Shanghai, China). The blank consisted of methanol.

For the liquid extract, five dilutions were prepared from Solution A (4000 mg/L), yielding concentrations of 500 mg/L, 248 mg/L, 124 mg/L, 60 mg/L, and 30 mg/L. After kinetic testing, a 22 min incubation time was established. Aliquots (100 μL) of each dilution (in duplicate) were mixed with 3.9 mL of DPPH radical solution. Absorbance was measured at 515 nm after 22 min. All procedures were performed in the dark. Results were expressed as g extract/g DPPH.

### 3.7. Cytotoxicity Investigation in Murine Macrophage Cells RAW 264.7

RAW 264.7 cells were grown in RPMI-1640 (Sigma Chemical Co., St. Louis, MO, USA) supplemented with 10% FBS (Cripion, São Paulo, Brazil). Cells were inactivated by incubation at 56 °C for 30 min in RPMI containing 2 mML-glutamine (Sigma Chemical Co.), 50 mM2-mercaptoethanol (Sigma-Aldrich), 100 U/mL penicillin, 100 mg/mL streptomycin (Sigma-Aldrich), and 2 mM Hepes (Sigma-Aldrich). Cells (2 × 10^5^) were grown in 2 mL of RPMI in a well of 6-well culture plates (Costar, New York, NY, USA) in a 37 °C incubator at 5% CO_2_. The RAW 264.7 cells (1 × 10^6^ cells/mL) were plated in triplicate in 96-well plates in RPMI-1640 medium supplemented with 10% FBS and treated with one of five concentrations of the *Eugenia involucrata* leaf extract (575, 1150, 2300, 4600, or 9200 µg/mL) dissolved in dimethyl sulfoxide solution (DMSO; 10 mg/mL). After 48 h of incubation, 5 mL of MTT (5 mg/mL) was added to each well, and the plates were incubated for an additional 3 h. The blue MTT formed a precipitate, which was then dissolved in DMSO. The absorbance was measured using a microplate reader (Biolisa Reader, Bioclin, Belo Horizonte, MG, Brazil) at 450 to 630 nm. Cell viability was expressed as a percentage of the control (not treated cells). Analysis of Variance (ANOVA) was used to compare treatments with the Tukey post-test (*p* < 0.05).

### 3.8. Determination of Cytotoxic and Genotoxic Activity in Human Lymphocytes

#### 3.8.1. Blood Samples

Human blood samples for in vitro cytotoxic and genotoxic evaluation were obtained from healthy volunteers (aged 18–30 years) with no history of chronic diseases, smoking, or alcoholism. The study was approved by the Research Ethics Committee of Universidade Estadual de Goiás (CEP No. CAAE 66860023.1.0000.8113).

#### 3.8.2. Human Lymphocyte Culture

Lymphocyte cultures were prepared using an adapted method from Rocha et al. (2020) [76]. Venous blood (20 mL) was collected in heparinized tubes, mixed with 20 mL RPMI medium, and centrifuged (120× *g*, 4 min, 18 °C). After plasma removal, the leukocyte layer was isolated using Ficoll-Paque PLUS (200× *g*, 10 min, 18 °C). Pellets were resuspended in RPMI medium supplemented with 10% fetal bovine serum, 0.5% phytohemagglutinin, and 0.2% penicillin–streptomycin antibiotic solution, then incubated (37 °C, 24 h) in a BOD incubator.

#### 3.8.3. Cytotoxicity and Cytoprotective Assay

Cell viability was assessed using 0.4% Trypan blue staining. Lymphocytes (4 × 10^5^ viable cells/well) in 24-well plates were treated with *E. involucrata* leaf extract (62.5–250 µg/mL in water) with/without 50 µg/mL doxorubicin (DXR) for 3 h (37 °C). Sterile water and DXR served as the negative and positive controls, respectively. Viable cells were recounted post-treatment. All tests were performed in triplicate.

#### 3.8.4. Genotoxicity and Genoprotective Effects (Comet Assay)

Treated lymphocytes (100 µL) were mixed with 120 µL low-melting-point agarose (1%), layered on agarose-coated slides, and lysed (Triton X-100 lysis buffer, 24 h). Electrophoresis was conducted in an alkaline buffer (300 mM NaOH, 4 °C, 25 V/cm, 30 min). Slides were neutralized (0.4 M Tris-HCl, pH 7.5), stained with Diamond™ Nucleic Acid Dye (1:10,000 in PBS), and analyzed via fluorescence microscopy (Axioplan-Imaging, 10× objective). DNA damage (% tail DNA) was quantified using CometScore™ (v1.5), with 100 cells analyzed per treatment. Statistical significance (*p* < 0.05) was determined by one-way ANOVA with Tukey’s test (GraphPad Prism 8).

## 4. Conclusions

The liquid extract of *Eugenia involucrata* leaves demonstrated the presence of phenolic compounds, namely, rutin and epicatechin. This extract holds significant importance due to its antioxidant effects, as evidenced by its iron-reducing capacity and free radical scavenging activity, which can lead to chemoprotective and antitumoral consequences. The liquid extract was not cytotoxic but was genotoxic to human lymphocytes at the highest tested concentration (250 µg/mL). Conversely, it demonstrated a genoprotective effect at the lowest tested concentration (62.5 µg/mL). This role as a protective agent against oxidative stress and a potential therapeutic alternative emphasizes the value of *Eugenia involucrata* in developing natural health products aimed at cancer prevention and treatment, thereby contributing to advancements in phytomedicine. Future perspectives include identifying the remaining compounds, conducting additional cytogenotoxic tests in systemic models (such as murine), and investigating its antitumoral potential in cancer cell cultures.

## Figures and Tables

**Table 1:** Antioxidant activity using the DPPH, FRAP, and ABTS^+^ methods on the liquid extract from the leaves of *Eugenia involucrata* DC. Capture of the free radical 2,2′-azinobis (3-ethylbenzothiazoline-6-sulfonic acid) (ABTS^+^); capacity to reduce the radical 2,2-diphenyl-1-picrylhydrazyl (DPPH); power of iron reduction (FRAP); relative standard deviation (%).

Antioxidant Activity	Mean	Coefficient of Variation
DPPH (g extract/g DPPH)	75.0	11%
FRAP (μM ferrous sulfate/g extract)	1490.79	1.2%
ABTS^+^ (μM Trolox/g extract)	2701.67	8%

**Table 2:** Gradient composition used as a mobile phase during phenolic compound screening by HPLC-DAD in the leaf material of *Eugenia involucrata*.

Time (min)	A (%)	B (%)
0–5	2	98
5–8	5	95
8–11	20	80
11–14	25	75
14–21	40	60
21–24	80	20
24–27	90	10
27–30	5	95
30–35	2	98

A: acetonitrile; B: 0.2% acetic acid in water (*v*/*v*).

## Data Availability

Data are contained within the article.

## Supplementary Materials

The following supporting information can be downloaded at https://www.mdpi.com/article/10.3390/ph18050764/s1: Figure S1. Image of the herbarium specimen deposited at the Herbarium of the State University of Goiás (code number 15366) of the *Eugenia involucrate* species. Figure S2. Chromatogram of the analytical standards at 280 nm (A), 306 nm (B), and 340 nm (C) by HPLC-DAD. Table S1. Cell viability (%) and standard deviation (SD) after 48 h of treatment of RAW 264.7 macrophages with the hydroalcoholic extract of *Eugenia involucrata* DC. Leaves.
