# Supplementary material for: Bioactive Compounds, Antioxidant, Cytotoxic, and Genotoxic Investigation of the Standardized Liquid Extract from Eugenia involucrata DC. Leaves
Source: Pharmaceuticals (Basel). 2025 May 21;18(5):764. doi: 10.3390/ph18050764 (PMC12114753; doi:10.3390/ph18050764)
Supplement: Supplementary file 1 [file pharmaceuticals-18-00764-s001.zip › pharmaceuticals-3618606-supplementary.pdf]

Supplementary material

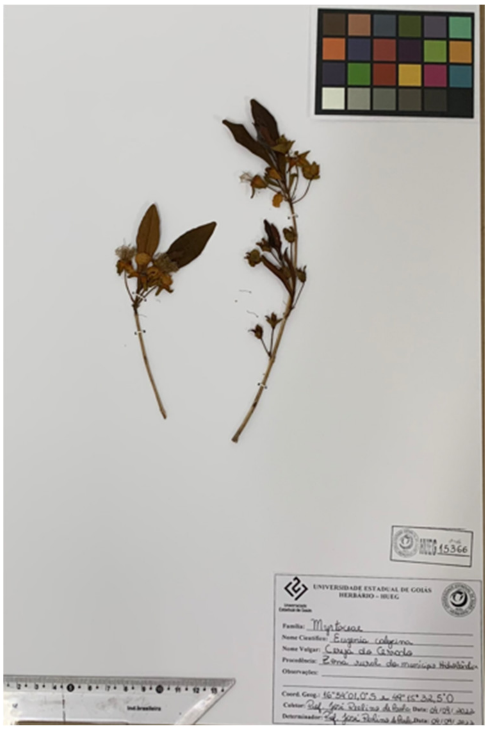

Figure S1. Image of the herbarium specimen deposited at the Herbarium of the State University of Goiás (code number 15366) of the *Eugenia involucrate* species. The label identifies it with the synonym name *Eugenia calycina*.

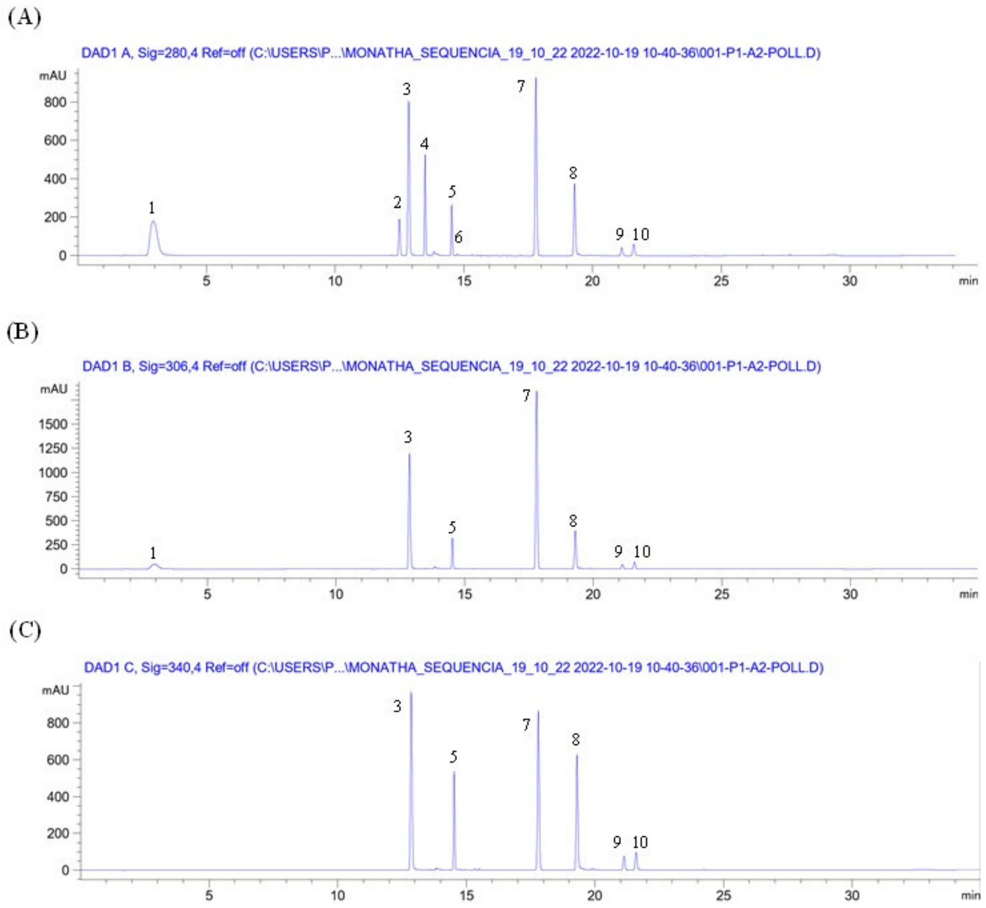

Figure S2. Chromatogram of the analytical standards at 280 nm (A), 306 nm (B), and 340 nm (C) by HPLC-DAD. Chromatographic conditions: The analysis was performed using a mobile phase consisting of acetonitrile and water acidified with 0.2% acetic acid, with gradient elution. The injection volume was 5 μL with a 1 mL/min flow rate, and the column temperature was maintained at 30°C. Detection was carried out using a diode array detector (DAD) with a C18 column. The following retention times (RT) were observed for the analyzed compounds: 1: gallic acid (RT: 2.936 min), 2: catechin (RT: 12.496 min), 3: caffeic acid (RT: 12.859 min), 4: epicatechin (RT: 13.495 min), 5: rutin (RT: 14.529 min), 6: ellagic acid (RT: 14.731 min), 7: resveratrol (RT: 17.800 min), 8: quercetin (RT: 19.303 min), 9: apigenin (RT: 21.134 min), and 10: kaempferol (RT: 21.602 min). Peak heights were measured in mAU, with all times reported in minutes (min) as retention times (RT).

Table S1. Cell viability (%) and standard deviation (SD) after 48 hours of treatment of RAW 264.7 macrophages with the hydroalcoholic extract of *Eugenia involucrata* DC. leaves.

Extract concentration (μg/mL)	Cell viability (%) ± SD
Control	100
575	146.51 ± 15.51
1150	62.95 ± 11.69
2300	24.79 ± 10.22
4600	29.14 ± 16.93
9200	14.47 ± 11.75

SD=Standard deviation.
